# Postoperative Showering for Patients With Closed Suction Drainage: A Retrospective Cohort Study of Deep Inferior Epigastric Perforator Flap Breast Reconstructions

**DOI:** 10.7759/cureus.23665

**Published:** 2022-03-30

**Authors:** Haruo Ogawa, Shinya Tahara

**Affiliations:** 1 Department of Plastic Surgery, Steel Memorial Hirohata Hospital, Himeji, JPN; 2 Department of Plastic Surgery, Meiwa Hospital, Nishinomiya, JPN

**Keywords:** surgical site infection, complication, shower, closed suction drainage, deep inferior epigastric perforator flap

## Abstract

Background

The deep inferior epigastric perforator (DIEP) flap has been widely used in breast reconstruction. During surgery, many surgeons use closed suction drainage for both the donor site and the reconstructed breast. However, the criteria for drainage removal depend on the surgeon’s preference and remain controversial. Moreover, it is well known that early postoperative showering is harmless to the surgical site and is recommended in many reports. However, it has not been discussed whether it is acceptable for patients with closed suction drainage to take a shower.

Methodology

We conducted a retrospective study of postoperative showering in 30 patients who underwent breast reconstruction with a DIEP flap. During the surgery, a total of three closed suction drains were connected to the patient’s body (one was connected to the reconstructed breast, and the other two were connected to the abdominal donor site). After the surgery, patients were allowed to shower when the number of connected drainage tubes was ≤2.

Results

The patients were divided into three groups according to the number of remaining drainage tubes connected to their bodies when they started postoperative showering. Group A included patients with no drainage tubes. Group B included patients with one remaining drainage tube. Group C included patients with two drainage tubes. No significant differences in the incidence of postoperative individual complications were observed among the three groups.

Conclusions

Postoperative showering for patients with closed suction drainage is safe and does not increase the incidence of postoperative complications, including surgical site infection.

## Introduction

The deep inferior epigastric perforator (DIEP) flap has been widely used for autologous breast reconstruction. In 2020, of the 137,808 patients who underwent breast reconstructive surgery in the United States, 23,324 underwent breast reconstruction with the DIEP flap [1]. In those cases, a majority of surgeons may have used postoperative closed suction drainage (CSD) for patients’ autologous donor sites to reduce dead space, blood, and serous fluid. Phillips et al. have reported that only 4.2% of surgeons have never used CSD in donor sites for autologous flap breast reconstruction [2].

When CSD is applied, drain removal criteria would be of interest to almost all surgeons. Most surgeons use a drainage volume threshold of ≤30 mL during a 24-hour period [2]. Liang et al. adopted a daily drainage volume of ≤40 mL on two consecutive days [3]. However, the criteria for daily drainage volume for drainage removal were set by the surgeon in a wide range, that is, from 5 mL to 80 mL [2]. Thus, the criteria for CSD removal vary as they depend on the surgeon’s preference.

Some authors have suggested the short-term use of CSD. Miranda et al. have reported that the removal of the donor site’s CSD early by postoperative day (POD) three has more significant advantages for patients than drain removal after POD three [4]. Moreover, they have reported no significant differences in postoperative complications between the two groups. Thacoor et al. have reported that drain-free abdominal closure in DIEP flap breast reconstruction could be safely achieved without increased postoperative complications [5]. No-drain techniques for DIEP flap donor site closure reportedly had a lower complication rate than the conventional abdominal drainage technique [6,7]. Meanwhile, a report has recommended the placement of CSD for breast reconstructions with a transverse rectus abdominis muscle (TRAM) flap [8]. Scevola et al. have stated that the incidence of postoperative seroma at the donor site of the TRAM flap was 2.1% when two drains were placed at the donor site and 7.1% when one drain was placed at the donor site, indicating a significant difference between the above-mentioned two groups [8]. This report described the incidence of seroma in the abdominal donor site due to breast reconstruction with a TRAM flap, not with a DIEP flap. However, this suggests that more CSD tubes may prevent postoperative seroma formation. Hence, the criteria of CSD placement and removal in the donor site of autologous breast reconstruction remain controversial.

Postoperative showers and bathing have also been widely discussed. According to the study by Phillips et al., 40.2% of surgeons restricted postoperative showering in patients who underwent autologous breast reconstructions [2]. However, their report did not provide details on the conditions for restriction and permission for postoperative showering. In contrast, many reports have recommended early postoperative showering in various surgical fields. Dayton et al. have conducted a systematic review of the influence of postoperative showering or bathing on the incidence of surgical site infection (SSI) [9]. They found that postoperative showering or bathing with tap water before suture or staple removal did not increase the incidence of SSI. Hsieh et al. have reported that wounds could be safely showered within 48 hours postoperatively [10]. Harrison has also identified no benefit in preventing patients from showering within the first 48 hours after the surgery compared to keeping the patient’s wound dry during the same period [11]. Because early postoperative showering can also bring comfort to patients, it can be more promoted if possible.

Meanwhile, the safety of postoperative showering and bathing for patients with CSD has not been reported. Hsieh et al. permitted the patients in their study to take a shower after CSD removal [10]. In other studies, the connection of CSD to the patient’s abdominal donor site when they started showering postoperatively has not been reported. Thus, this study aimed to investigate whether patients can shower with a CSD connected to the site.

## Materials and methods

Study design

A single-center, single-surgeon retrospective study was performed from January 2018 to February 2021 at the Japanese Red Cross Kobe Hospital/Hyogo Emergency Medical Center, Kobe, Japan. Patients who underwent autologous breast reconstruction using the DIEP flap were included. Patients who received preoperative or postoperative radiation therapy or chemotherapy were excluded from the study. Patients who had diabetes, connective tissue disease, immunodeficiency, liver disease, or renal disease as well as those considered to be compromised hosts were also excluded from the study. Patient data including age, preoperative nutritional status, body mass index (BMI), intraoperative bleeding volume, usage of CSD, and postoperative complications at the abdominal donor site were collected. All procedures used in this research were approved by the Ethical Committee of the Japanese Red Cross Kobe Hospital/Hyogo Emergency Medical Center (permission number: 255). Written informed consent was obtained from all patients.

Surgical procedure and postoperative care

After a mastectomy or after the creation of a breast footprint, the DIEP flap was harvested by the first author. The abdominal donor site was closed using the following procedure. First, after confirming hemostasis, the rectus sheath was closed with absorbable sutures (STRATAFIX® Symmetric PDS PLUS®, Ethicon, New Brunswick, NJ, USA). Second, CSD tubes were placed on the sutured rectus sheath. Third, the skin and subcutaneous tissue were closed layer by layer. Quilting sutures were not applied for abdominal donor site closure.

After the surgery was completed and the general anesthesia’s effects wore off, patients were transferred to the intensive care unit. After spending one night in the intensive care unit, patients were transferred to the general ward. On POD one, patients were allowed to walk in the general ward, and movement restriction was gradually reduced depending on the patients’ motivation and general condition. Postoperative antibiotics were administered to all patients until POD two. The sutured wound was covered with dressing material (Aquacel® Surgical, ConvaTec, Reading, UK) until suture removal on POD six.

Usage of CSD

**Figure 1:**
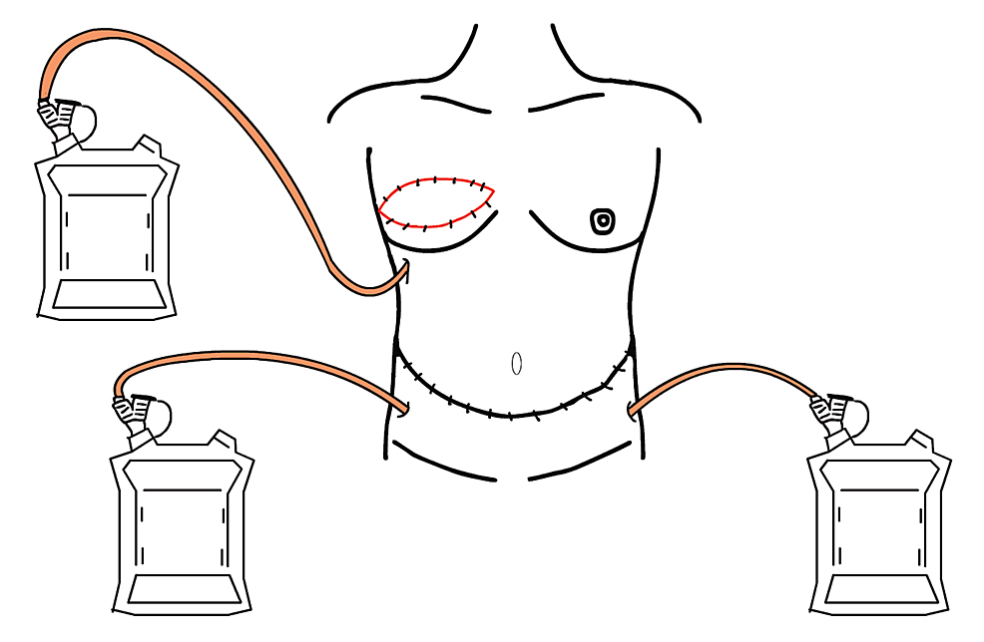
The setting of the closed suction drainage tube on the patient’s abdominal donor site. One drainage tube was connected to the reconstructed breast subcutaneously. Two drainage tubes were connected to the patient’s abdominal donor site from each side. Image credits: Haruo Ogawa.

In all patients who underwent breast reconstructive surgery with the DIEP flap, J-VAC® (150 mL, Ethicon, New Brunswick, NJ, USA) and Blake® (5 mm, Ethicon, New Brunswick, NJ, USA) were used as CSD materials. Before wound closure, a drainage tube was placed subcutaneously in the reconstructed breast. In the abdominal donor site, two drainage tubes were placed on the rectus sheath subcutaneously; one drainage tube was connected from the right lower lateral abdomen, and the other was connected from the left lower lateral abdomen. J-VAC was attached to each tube separately (Figure 1).

**Figure 2:**
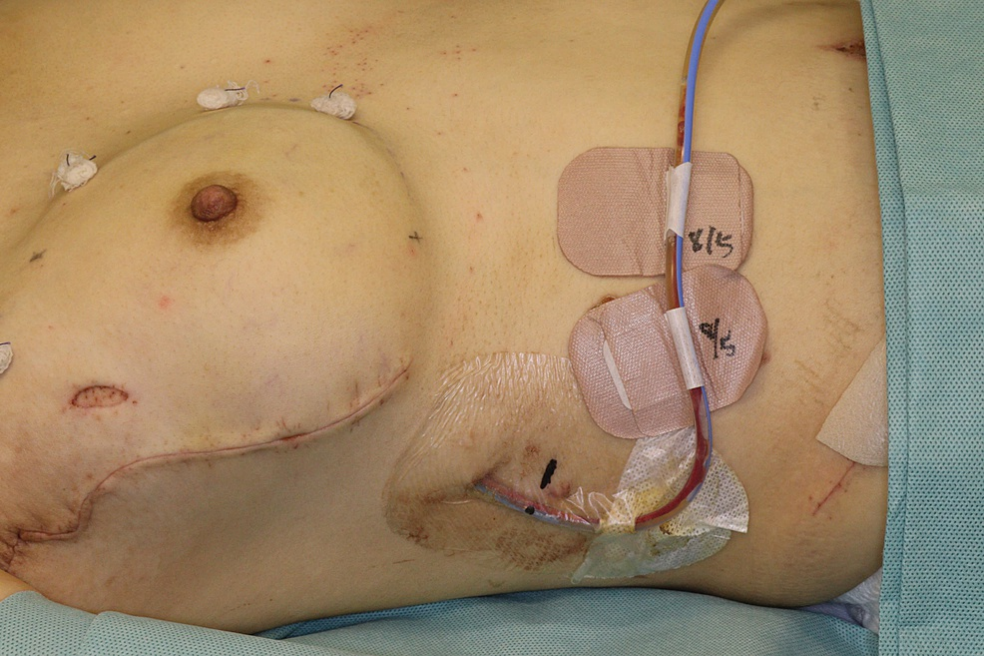
The condition of the closed suction drainage tube dressing. The insertion site of the drainage tube was covered with IV3000 ported. However, the drain insertion sites were not completely waterproofed.

The insertion site of the Blake drain tube into the patient’s body was covered with IV3000 ported (Smith & Nephew, Watford, UK) at the end of the breast reconstruction surgery (Figure 2).

However, the insertion site of the Blake drain tube was not completely waterproofed in all cases. This is because there was always a gap between the drain tube and the IV3000 ported. Moreover, we did not take any action to close this gap completely. When the patient started postoperative showering, the IV3000 ported remained attached to the drainage tube insertion site, with the insertion site of the drain tube not waterproofed. Not only surgeons but also clinical staff in the patient’s ward and the patients understood that the CSD insertion sites were not completely waterproofed. While the patient was taking a shower, the J-VAC and Blake were secured by the clinical staff so that they would not fall out of the patient’s body. The criteria for drainage removal were that, in each J-VAC, the drainage fluid turned serous, and the amount of drainage was ≤35 mL during a 24-hour period. The authors used this criterion habitually for postoperative closed suction drainage use.

Criteria for permitting postoperative showering

Patients who were judged to be able to walk safely in the general ward and had no more than two CSD tubes connected to the abdominal donor site were permitted to take a shower with the assistance of the hospital staff. If the patient did not wish to shower with the CSD tube connected to the abdominal donor site, a postoperative shower was initiated at the patient’s desired period. All cases were divided into three groups according to the number of CSD tubes connected to the abdominal donor site at the start of the postoperative shower. In group A, patients started postoperative showering after all CSD tubes had been removed. In group B, the patients started postoperative showering with one remaining CSD tube connected to the abdominal donor site. In group C, the patients started postoperative showering with two CSD tubes connected to the abdominal donor site.

Statistical analysis

All data are presented as average, standard deviation (SD), minimum value, maximum value, and median. The data were analyzed using EZR (Saitama Medical Center, Jichi Medical University, Saitama, Japan), which is a graphical user interface for the R software (The R Foundation for Statistical Computing, Vienna, Austria). It is a modified version of the R Commander designed to add statistical functions frequently used in biostatistics [12].

Analysis of BMIs among the groups was examined using a nonparametric multiple comparison test (Kruskal-Wallis test followed by Steel-Dwass post hoc test for the analysis) because the BMIs in group A and group C did not show the normal distribution even if the logarithmic conversion was performed. Regarding the start date of postoperative showering and the day of final CSD tube removal, we also conducted a nonparametric multiple comparison test (Kruskal-Wallis test followed by Steel-Dwass post hoc test for the analysis) because the data in each group did not show a normal distribution. The age, nutritional status, and intraoperative bleeding volume were analyzed using the one-way analysis of variance test, followed by the Tukey post-hoc test for the analysis. Regarding the incidence of postoperative complications among the three groups, variables were analyzed using Fisher’s exact test, followed by Holm and Bonferroni post-hoc tests for the analysis. Statistical significance was set at p-values of <0.05.

## Results

Here, 30 patients who underwent DIEP flap breast reconstruction were recruited. Of the 30 patients, 29 underwent one-sided breast reconstruction, and one underwent bilateral breast reconstruction. According to the number of vascular pedicles in one DIEP flap, six cases had a double pedicle, and 24 cases had a single vascular pedicle.

**Figure 3:**
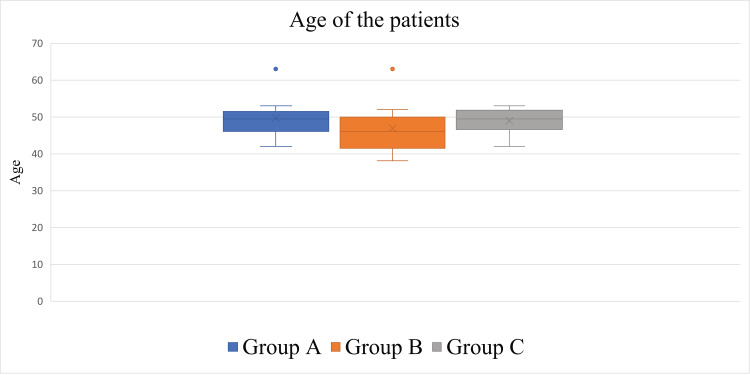
Box plots showing the ages of the groups. No significant differences were observed among the groups (p = 0.33).

The average age of patients and the SD were 48.30 ± 5.61 years (38-63, median = 49). The average and SD of the ages in each group were as follows: 49 ± 5.39 (42-63, median = 49.5) years in Group A, 46.83 ± 6.39 (38-63, median = 46) years in Group B, and 48.88 ± 3.33 (42-53, median = 49.5) years in Group C. No significant differences in ages were observed among the groups (p = 0.33) (Figure 3, Table 1).

**Table 1:** The comparison among the groups. Mean ± SD and median (minimum–maximum) for continuous variables are shown. *One-way ANOVA followed by Tukey post hoc test was conducted for the analysis. **Kruskal-Wallis test followed by Steel-Dwass post hoc test was conducted for the analysis. BMI: body mass index; POD: postoperative day; SD: standard deviation; ANOVA: analysis of variance

	Group A	Group B	Group C	Total	P-value
Number of patients	10	12	8	30	
Age	49.6 ± 5.39	46.83 ± 6.39	48.88 ± 3.33	48.30 ± 5.61	0.33*
	49.5 (42–63)	46 (38–63)	49.5 (42–53)	49 (38–63)	
BMI	21.93 ± 2.25	20.98 ± 2.65	21.85 ± 3.78	21.52 ± 2.81	0.61**
	21.24 (19.65–26.16)	20.33 (18.21–28.36)	21.28 (18.17–27.95)	20.79 (18.17–28.36)	
Nutritional status (serum albumin)	4.14 ± 0.20	4.13 ± 0.23	4.04 ± 0.24	4.11 ± 0.22	0.59*
	4.1 (3.9–4.6)	4.1 (3.8–4.5)	3.95(3.8–4.5)	4.1 (3.8–4.6)	
Intraoperative bleeding volume	154.5 ± 87.13	169.17 ± 94.36	158.75 ± 73.76	161.5 ± 82.79	0.92*
	140 (50–350)	147.5 (30–320)	175 (50–250)	160 (30–350)	
Start date of postoperative showering (POD)	6.80 ± 2.94	4.92 ± 1.31	4.63 ± 1.19	5.47 ± 2.15	0.20**
	7 (4–13)	5 (3–8)	4.5 (3–7)	5 (3–13)	
Postoperative duration before drain removal (days)	6.10 ± 2.33	7.33 ± 1.87	8.25 ± 2.25	7.17 ± 2.23	0.08**
	6(4–11)	7 (5–11)	8 (5–12)	7 (4–11)	

**Figure 4:**
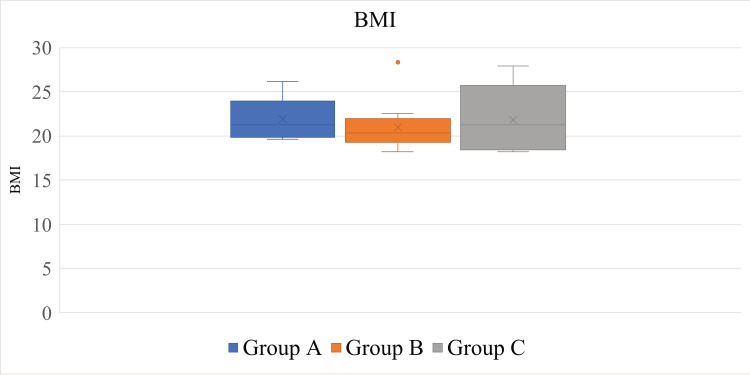
Box plots of the BMIs of the groups. No significant differences were observed among the groups (p = 0.61).

The average BMI and SD of the patients were 21.52 ± 2.81 (18.17-28.36, median = 20.79) kg/m^2^. The average and SD of the BMIs in each group were as follows: 21.93 ± 2.25 (19.65-26.16, median = 21.24) kg/m^2^ in Group A, 20.98 ± 2.65 (18.21-28.36, median = 20.33) in Group B, and 21.85 ± 3.78 (18.17-27.95, median = 21.28) kg/m^2^ in Group C. No significant differences in the BMIs were observed among the groups (p = 0.61) (Figure 4, Table 1).

**Figure 5:**
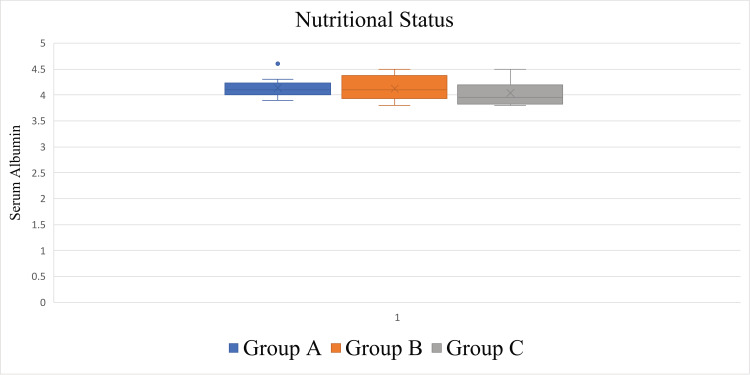
Box plots showing the nutritional status of the groups. No significant differences were observed among the groups (p = 0.59).

The average nutritional status (serum albumin) of patients and the SD was 4.11 ± 0.22 (3.8-4.6, median = 4.1) g/dL. The average and SD of serum albumin in each group were as follows: 4.14 ± 0.20 (3.9-4.6, median = 4.1) g/dL in Group A, 4.13 ± 0.23 (3.8-4.5, median = 4.1) g/dL in Group B, and 4.04 ± 0.24 (3.8-4.5, median = 3.95) g/dL in Group C. No significant differences in the nutritional status were observed among the groups (p = 0.59) (Figure 5, Table 1).

**Figure 6:**
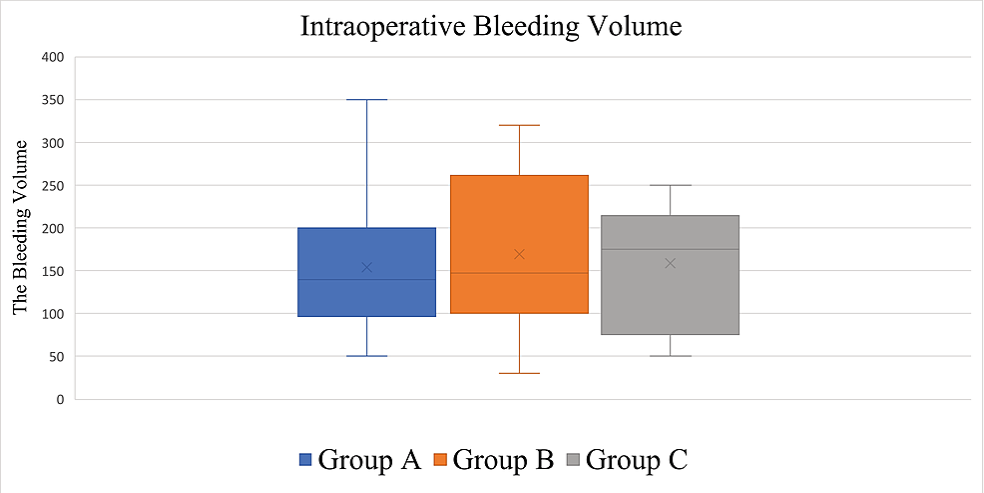
Box plots showing intraoperative bleeding volumes. No significant differences were observed among the groups (p = 0.92).

The average intraoperative bleeding volume of the patients and the SD were 161.50 ± 82.79 (30-350, median = 160) mL. The average and SD of the intraoperative bleeding volume in each group were as follows: 154.50 ± 87.13 (50-350, median = 140) mL in Group A, 169.17 ± 94.36 (30-320, median = 147.5) mL in Group B, and 158.75 ± 73.76 (50-250, median = 175) mL in Group C. No significant differences in the nutritional status were observed among the groups (p = 0.92) (Figure 6, Table 1).

All patients were able to walk in the general ward by POD three.

Postoperative showering and CSD removal

The average start date of postoperative showering and SD in all cases was POD 5.47 ± 2.15 (3-13, median = 5). In addition, all CSD tubes were removed on average on POD 7.17 ± 2.23 (4-12, median = 7).

**Figure 7:**
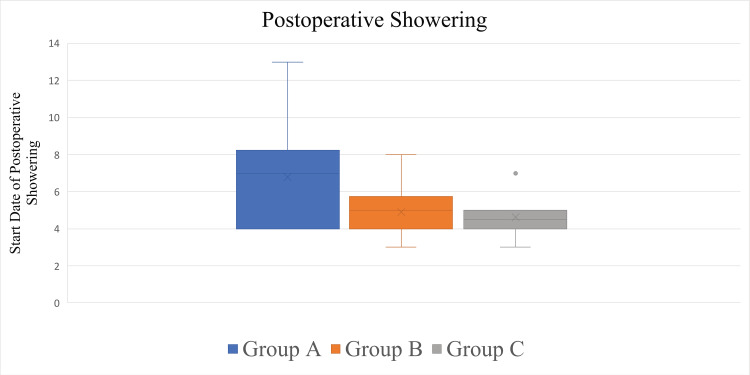
Box plots showing distributions of the start dates of the postoperative showering. No significant differences were observed among the groups (p = 0.20).

Groups A, B, and C comprised 10, 12, and 8 patients, respectively. The respective average dates and SDs when the patients started postoperative showering were POD 6.80 ± 2.94 (4-13, median = 7) in Group A, POD 4.92 ± 1.31 (3-8, median = 5) in Group B, and POD 4.63 ± 1.19 (3-7, median = 4.5) in Group C (Figure 7, Table 1).

**Figure 8:**
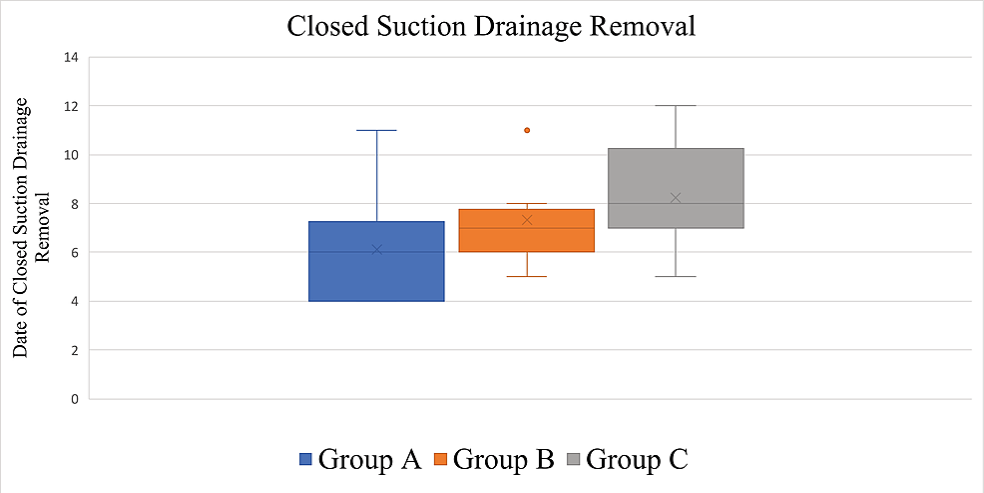
Box plots showing distributions of the dates of the closed suction drainage removal. No significant differences were observed among the groups (p = 0.08).

The respective average dates and SDs when all CSD tubes were removed from the abdominal donor site were POD 6.10 ± 2.33 (4-11, median = 6) in Group A, POD 7.33 ± 1.87 (5-11, median = 7) in Group B, and POD 8.25 ± 2.25 (5-12, median = 8) in Group C (Figure 8, Table 1). The statistical analysis revealed no significant differences between the groups on the day the patients started postoperative showering (p = 0.20).

On the day when all CSD tubes were removed from the abdominal donor site, the Kruskal-Wallis test indicated no significant differences among the three groups (p = 0.08). The Steel-Dwass post hoc test showed the following results: Group A-Group B: p = 0.38; Group A-Group C: p = 0.10; Group B-Group C: p = 0.36.

Comparison of the incidence of postoperative abdominal complications among the three groups

Of the 30 patients, four patients experienced postoperative complications in the abdominal donor site. SSI at the abdominal donor site occurred in two patients in Group B; one patient had SSI on POD eight, and the other on POD 10. The SSI sites of both patients were on the central lower abdomen just caudal to the sutured wound of the donor site. They were very close to the site where the drain tube was placed in the left abdomen. The first patient, whose intraoperative bleeding volume was 145 mL, started postoperative showering on POD four. The patient’s abdominal drainage was removed on POD seven. On POD eight, redness and swelling occurred around the midline of her lower abdomen just caudal to the DIEP flap donor site. On POD 10, wound dehiscence occurred in the central part of the donor site and the pus was drained. *Serratia marcescens* was detected as a result of bacterial examination from the wound. Ceftriaxone sodium hydrate (2 g/day) administration was begun and the wound was cleaned daily. The patient recovered from the SSI and the wound was closed on POD 16. The other patient, whose intraoperative bleeding volume was 300 mL, started postoperative showering on the same day after the removal of one abdominal drainage tube on POD eight. Redness and swelling occurred in her lower abdomen just caudal to the central part of the donor site on POD 10. At the same time, wound dehiscence occurred in her umbilical wound. The second drainage was removed on POD 11. On POD 14, methicillin-resistant *Staphylococcus aureus* (MRSA) was detected as a result of bacterial examination from the wound. From that day on, the donor site and umbilical wound cleaning was performed daily and vancomycin hydrochloride (2 g/day) administration was started. The SSI subsided and the wound was closed on POD 18. In these patients, the infections were confined to the skin and subcutaneous tissue and did not extend to the deep tissue including rectus sheath or rectus abdominis muscle. Therefore, these SSIs were considered to be superficial incisional SSIs.

Additionally, hemorrhage occurred in one case in Group B. The patient who developed postoperative hemorrhage in her abdominal donor site started postoperative showering on POD six. The remaining drainage tube was finally removed on POD eight, and she was discharged on POD 11. On POD 14, she was admitted to our department because of a sudden bulge in her abdomen. Computed tomography revealed a mass suspected to be a hematoma in the subcutaneous abdomen, and a hematoma removal surgery was performed. Subsequently, no postoperative complications were observed in the abdominal donor site. Postoperative complications that occurred at the abdominal donor site were examined (Table 2).

**Table 2:** Postoperative complications in abdominal donor site in each group. ***Fisher’s exact test followed by Holm and Bonferroni post hoc tests were conducted for the analysis. Significant differences in the incidence of any type of postoperative abdominal complications (p = 0.03). However, the post hoc test revealed no significant differences between each of the two groups. No significant differences were identified in the incidence of individual postoperative abdominal complications among all the groups. SSI: surgical site infection

	Group A	Group B	Group C	P
SSI	0	2	0	0.32***
Hematoma	0	1	0	1.00***
Seroma	0	1	0	1.00***
All Complications	0	4	0	0.03***

Significant differences were noted in the incidence of any type of postoperative abdominal complications (p = 0.03). However, the post hoc test revealed no significant differences between each of the two groups (Bonferroni post hoc test, P(A-B) = 0.29; P(A-C) = 1.00; P(B-C) = 0.35; Holm post-hoc test, P(A-B) = 0.29; P(A-C) = 1.00; P(B-C) = 0.29). No significant differences were identified in the incidence of individual postoperative abdominal complications (SSI, p = 0.32; hematoma, p = 1.00; seroma, p = 1.00) among all groups.

## Discussion

Our study demonstrated some interesting results regarding postoperative showering in patients with CSD after DIEP flap breast reconstruction. First, no significant differences were observed between the number of CSD tubes connected to the abdominal donor site and the incidence of postoperative complications. Therefore, we believe that postoperative showering does not increase the risk of postoperative complications in patients. Second, the number of CSD tubes connected to the abdominal donor site at the time the patient started the postoperative shower did not make a difference in the timing of CSD removal among the three groups. We believe that postoperative showering in patients with CSD would not delay the period of final CSD removal. Third, no significant differences in the period of starting postoperative showering were identified among the three groups. Before the investigation, we hypothesized that patients could start postoperative showering early if they were allowed to take a shower with more CSD tubes connected to the abdominal donor site. For example, we hypothesized that patients with two CSD tubes can take a shower earlier than patients with one CSD tube. Similarly, we also hypothesized that patients with one CSD tube can take a shower earlier than patients without a CSD tube. However, the results did not comply with our hypothesis. Comparing the mean values, Group A, in which the patient started showering after all CSDs were removed, appeared to be late in starting the postoperative shower. However, the comparison of mean values is not helpful because the variables among the groups did not show normal distributions. As mentioned earlier, no significant difference was noted among the groups for the start date of the postoperative showering. The Steel-Dwass post hoc test showed the following results: Group A-Group B: p = 0.38, Group A-Group C: p = 0.10, Group B-Group C: p = 0.36. Compared to other comparisons, there may be a tendency of the difference between Groups A and B. Because the sample sizes of the study were small and the start of the postoperative showering depended on the patient’s preference, such a result might occur.

To date, no reports of postoperative showering with CSD connected to a patient’s abdominal donor site exist. In a review on postoperative showering, Dayton et al. did not mention whether the patients were allowed to shower or not while the patients had a CSD connected to their donor site [9]. Meanwhile, some studies have reported the avoidance of postoperative showering while with a CSD connection. In the report by Hsieh et al., patients started postoperative showering after CSD removal [10], although the reason for this was not stated. Gök et al. noted that the sternal incision site was never exposed to water after coronary artery bypass graft surgery until the removal of the chest tube sutures according to their institution’s standards [13]. This suggests that their patients were not allowed to shower while the chest tube was connected to the patient’s body. Therefore, we believe that many surgeons are apprehensive of increased complication rates from patient showering during CSD installation and retrograde infection from the CSD insertion site.

Meanwhile, many reports have recommended patients showering during the early postoperative stage. Moreover, Gök et al. stated that early postoperative showering after sternotomy for coronary artery bypass graft surgery was protective against sternal wound infections [13]. They have also stated that the patients who took a shower within 48-72 hours postoperatively had significantly lower pain scores and higher comfort and satisfaction scores than the patients who did not take a shower in the early postoperative period [13]. Hsieh et al. have revealed that postoperative wounds can be safely showered 48 hours after surgery [10]. Harrison has noted that postoperative showering within the first 48 hours has no evidence of increasing SSI incidence and might improve patient comfort [11]. Thus, early postoperative showering before suture removal is recommended in many surgical fields.

In all our cases, the CSD tubes in all patients were removed from the abdominal donor site at POD 7.17 days on average (SD = 2.23, minimum = 4, maximum = 11, and median = 7). Moreover, all CSD tubes in Group A patients, who started postoperative showering after all CSD removal, could be completely removed at POD 6.80 days on average (SD = 2.94, minimum = 4, maximum = 13, and median = 7). If all patients would be prohibited to start postoperative showering until all the CSD tubes have been removed, the patients would not be able to shower for approximately one week postoperatively. Miranda et al. have recommended early drain removal by POD three [4]. Even in this situation, the patient may not be able to take a shower for three days postoperatively. In contrast, Gök et al. and Harrison et al. have suggested a much earlier start of postoperative showering than the PODs of the CSD removal, as recommended by Miranda et al. Therefore, there is a contradiction between the idea of starting early postoperative showering and starting showering after CSD removal. Therefore, considering the availability of postoperative showering for patients with CSD is extremely important.

In this study, starting postoperative showering while with a CSD in patients who underwent DIEP flap breast reconstruction did not cause more postoperative complications. Moreover, starting postoperative showering in patients with CSD did not delay the removal of all CSD tubes. In our cases, one hemorrhage, one seroma, and two SSIs occurred as postoperative complications in the abdominal donor sites. All patients who experienced complications belonged to Group B. Meanwhile, no patients developed postoperative complications in Group C despite taking a shower while having more CSD tubes than those in Group B. Moreover, the statistical analysis revealed no significant differences in the incidence of individual abdominal complications among the three groups. Thus, if the number of drains connected to the abdominal donor site was ≤2, the patient could safely take a shower regardless of drainage attachment.

The question remains as to why postoperative complications occurred only in the patients in Group B. None of the patients deviated significantly from the population in age, BMI, and nutritional status. The latter of the patients who developed SSI had an intraoperative bleeding volume of 300 mL. This bleeding volume was the second highest in Group B. However, the remaining three patients did not have any obvious cause of postoperative complications.

Further, no significant difference was observed among the three groups when postoperative showering was started. Initially, we hypothesized that groups in which patients had more CSD tubes connected to the abdominal donor site would start postoperative showering much earlier than those who have ≤1 CSD tube. Therefore, these results disagree with our expectations.

There are some limitations to our study. The abdominal donor site’s wound was covered with dressing material at the end of the operation. The donor site suture was regularly removed on POD six. Therefore, the condition of the abdominal donor site differed depending on when the patient started postoperative showering. However, many authors have stated that showering the sutured wound in the early postoperative period did not cause hazardous complications to the patient. Therefore, we believe that the differences in the conditions of each patient’s abdominal donor site at postoperative showering did not significantly affect our results. Another limitation is the use of perioperative antibiotics. There is no evidence-based research regarding the proper indication and duration of perioperative antibiotic use. According to Phillips et al., 46% of surgeons preferred concomitant discontinuation of antibiotic use with CSD use, whereas 52% of surgeons preferred a specific POD [2]. Similarly, with the latter study, we continued postoperative antibiotic use until POD two. Here, four of the 30 patients had postoperative complications, and of the recorded postoperative complications, three were abdominal donor site complications. Regarding the postoperative SSI rate, two of 21 patients developed postoperative SSI at the donor site of the DIEP flap. Garvey et al. reported an 11.5% donor site infection rate for the DIEP flap [14]. The incidence of postoperative complications in our cases was not higher than that in other reports [4,5,14]. Therefore, we do not believe that antibiotic use had a significant impact on our results. This study has a small sample size. Moreover, patients who received radiation therapy and chemotherapy, or those who were considered compromised hosts, were excluded from the study. These may be also limitations of the study.

## Conclusions

We investigated the safety of postoperative showering in patients with CSDs after autologous breast reconstruction surgery. Among the three groups, no significant differences in the incidence of postoperative individual complications were observed. Our findings indicate that postoperative showering is safe in patients in whom a CSD is connected after DIEP flap breast reconstruction. Additional or further research to confirm the safety of postoperative showering in the wide range of patients who have undergone DIEP flap breast reconstruction is needed in the future, in view of the smaller sample size of our study and exclusion of immunocompromised hosts.

## References

[1] (2022). American Society of Plastic Surgery: reconstructive demographics. http://www.plasticsurgery.org/documents/News/Statistics/2020/reconstructive-procedure-demographics-2020.pdf.

[2] Phillips BT, Wang ED, Mirrer J, Lanier ST, Khan SU, Dagum AB, Bui DT (2011). Current practice among plastic surgeons of antibiotic prophylaxis and closed-suction drains in breast reconstruction: experience, evidence, and implications for postoperative care. Ann Plast Surg.

[3] Liang DG, Dusseldorp JR, van Schalkwyk C, Hariswamy S, Wood S, Rose V, Moradi P (2016). Running barbed suture quilting reduces abdominal drainage in perforator-based breast reconstruction. J Plast Reconstr Aesthet Surg.

[4] Miranda BH, Amin K, Chana JS (2014). The drain game: abdominal drains for deep inferior epigastric perforator breast reconstruction. J Plast Reconstr Aesthet Surg.

[5] Thacoor A, Kanapathy M, Torres-Grau J, Chana J (2018). Deep inferior epigastric perforator (DIEP) flap: impact of drain free donor abdominal site on long term patient outcomes and duration of inpatient stay. J Plast Reconstr Aesthet Surg.

[6] Chan SL, Rutherford C, Kong TY (2020). No-drain technique in abdominal closure for breast reconstruction: lower complication rate, shorter hospitalization stay. Plast Reconstr Surg Glob Open.

[7] Nagarkar P, Lakhiani C, Cheng A, Lee M, Teotia S, Saint-Cyr M (2016). No-drain DIEP flap donor-site closure using barbed progressive tension sutures. Plast Reconstr Surg Glob Open.

[8] Scevola S, Youssef A, Kroll SS, Langstein H (2002). Drains and seromas in TRAM flap breast reconstruction. Ann Plast Surg.

[9] Dayton P, Feilmeier M, Sedberry S (2013). Does postoperative showering or bathing of a surgical site increase the incidence of infection? A systematic review of the literature. J Foot Ankle Surg.

[10] Hsieh PY, Chen KY, Chen HY (2016). Postoperative showering for clean and clean-contaminated wounds: a prospective, randomized controlled trial. Ann Surg.

[11] Harrison C, Wade C, Gore S (2016). Postoperative washing of sutured wounds. Ann Med Surg (Lond).

[12] Kanda Y (2013). Investigation of the freely available easy-to-use software 'EZR' for medical statistics. Bone Marrow Transplant.

[13] Gök F, Demir Korkmaz F, Emrecan B (2022). The effects of showering in 48-72 h after coronary artery bypass graft surgery through median sternotomy on wound infection, pain, comfort, and satisfaction: randomized controlled trial. Eur J Cardiovasc Nurs.

[14] Garvey PB, Buchel EW, Pockaj BA, Casey WJ 3rd, Gray RJ, Hernández JL, Samson TD (2006). DIEP and pedicled TRAM flaps: a comparison of outcomes. Plast Reconstr Surg.

